# Mapping spatial locational trends of informal economic enterprises using mobile geographic information data in the city of in Harare, Zimbabwe

**DOI:** 10.1016/j.dib.2018.09.037

**Published:** 2018-09-18

**Authors:** Trynos Gumbo, Manie Geyer, Inocent Moyo, Thembani Moyo

**Affiliations:** a Town and Regional Planning Department, University of Johannesburg, South Africa; b Centre for Regional Urban and Statistical Exploration (CRUISE), Stellenbosch University, South Africa; c Department of Geography and Environmental Studies, University of Zululand, South Africa; d Department of Operations and Management, University of Johannesburg, South Africa

**Keywords:** Mobile GIS, Spatial location, Informal economy, Harare, Zimbabwe

## Abstract

Spatial planning for informal economic enterprises globally and cities of the developing world such Harare in particular is made difficult by the lack of appropriate data. In most cases, informal economic enterprises are discussed descriptively and statistically, leaving out their spatial characteristics. This makes the orderly planning for the enterprises very difficult if not impossible, especially given that the informal economy dominates the economies of most developing countries. This article presents geographic information data that was collected by means of mobile geographic positioning systems over time. In the absence of any other spatial datasets in the City of Harare, this unique data is handy in revealing spatial locational trends of informal economic enterprises and the preferred locational behaviour of informal economic entrepreneurs in the city.

**Specifications table**

**Table:** 

**Subject area**	**Economic geography**
**More specific subject area**	Urban informality
**Type of data**	Maps, table and figures
**How data was acquired**	Field survey
**Data format**	Analysed
**Experimental factors**	Checking and arrangement of way points to ensure accuracy
**Experimental features**	Georeferencing of shapefiles, importing of waypoints and spatial analysis
**Data source location**	Harare, Zimbabwe, 17.8252°S, 31.0335°E
**Data accessibility**	Data is with this article
**Related research article**	Moyo I, Nicolau M. D and Gumbo T (2016) Johannesburg (South Africa) Inner City African Immigrant Traders: Pathways from Poverty? Urban Forum, pp 1–17 [1]
	Gumbo T. and Geyer H.S (2011) ‘Picking up the Pieces’: Reconstructing the informal economic sector in Bulawayo, Zimbabwe, Journal of Town and Regional Planning, 59, pp 53–64 [2]

**Value of the data**

• The GIS data is useful in that, for the first time the spatial locational trends of informal economic enterprises have been illustrated for the city of Harare.
• The GIS data and maps are useful to other researchers in Harare and elsewhere in cities of the developing world who have had no opportunity to access spatial data on the location and spread of informal economic enterprises overtime.
• The GIS data can be used to conduct spatial planning by city practitioners as they seek to ensure orderly management of informal economic enterprises in most cities of the developing countries where the phenomenon is prevalent.

## 1 Data

The data presented herein shows the spatial location and spread of informal economic enterprises (IEEs) in Harare over time. The locations of the IEEs are categorised into the Central business districts (CBD); Suburban Shopping Centre (SSCs); Industrial centres (ICs); Transportation and Communication Centres (TCs) and Homes/open spaces and Roads (HB). The types of the IEEs are divided into Trade and commerce; Manufacturing and processing; Personal Services; Transport and communication and construction and development. The dominance of the different types of the IEEs at various locations is demonstrated by the data.

### 1.1 Locational spread and rising informal economic enterprises

**Fig. 1:**
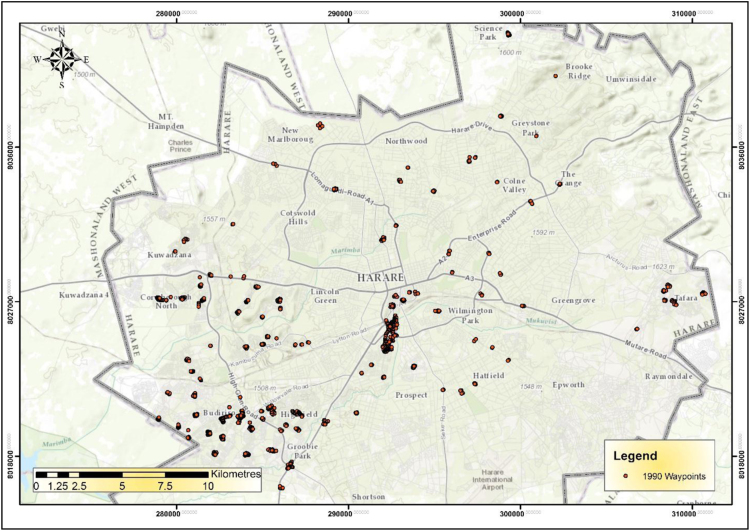
Waypoints in Harare for the year 1990.

**Fig. 2:**
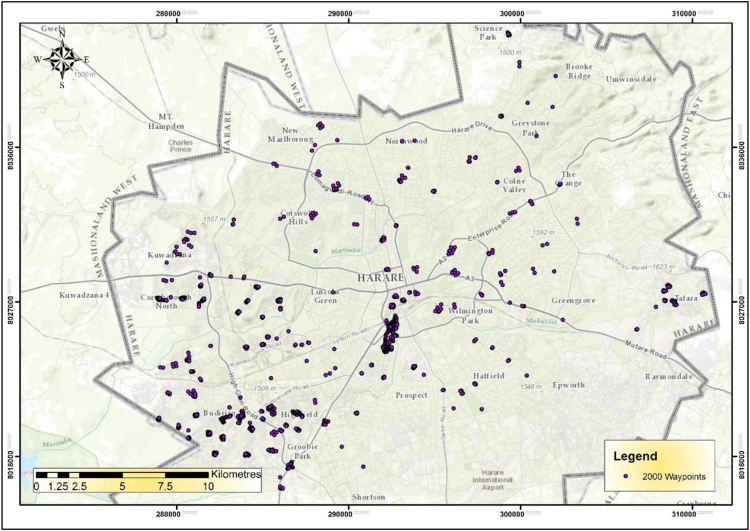
Waypoints in Harare for the year 2000.

**Fig. 3:**
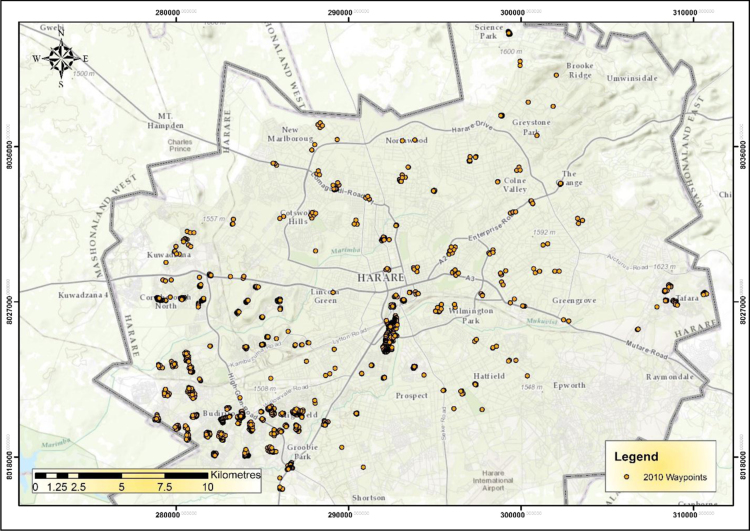
Waypoints in Harare for the year 2010.

**Fig. 4:**
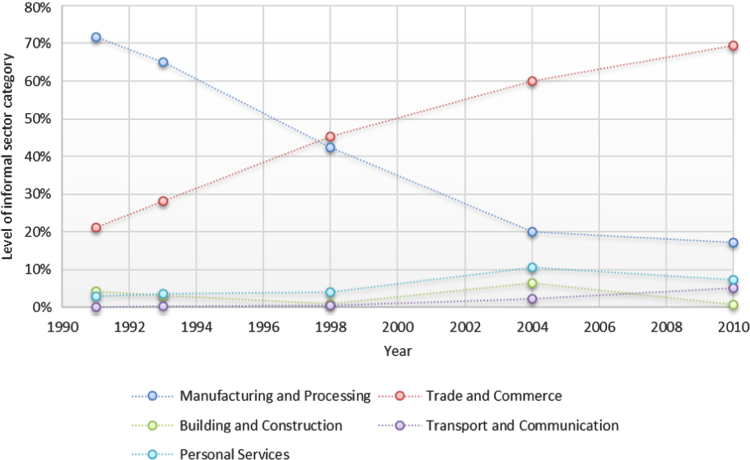
Informal economic enterprises in Harare by categories from 1990 to 2010. (Source: [2], [3])

The IEEs, particularly trade and commerce were largely concentrated within the CBD and low income and poor residential neighbourhoods by 1990 (Fig. 1). The trend started changing during the 1990s as the country experienced structural economic changes [3], [4], [5], [6]. Consequently, by 2000 the IEEs had not only changed in type and character but also in operating places as they had invaded the high income residential areas (Fig. 2). At the beginning of the new millennium, the IEEs expanded and started spreading into Industrial centres (ICs); Suburban Shopping Centre (SSCs); Transportation and Communication Centres (TCs) and concentrating more on the CBD and residential areas of all types in order to gain the market (Fig. 3). The IEEs soared sharply during the 20-year period in Harare (Table 1; Fig. 4).

**Table 1:** Informal economic enterprises in Harare by categories from the years 1990 to 2010.

Business category	**1990**	**2000**	**2010**
Trade and commerce	106,678	256,982	431,279
Manufacturing and processing	43,028	117,632	136,840
Personal services	9387	21,754	27,598
Transport and communication	23,729	52,098	73,902
Construction and development	11,937	19,652	24,276
Total	194,759	468,118	693,895

**Fig. 5:**
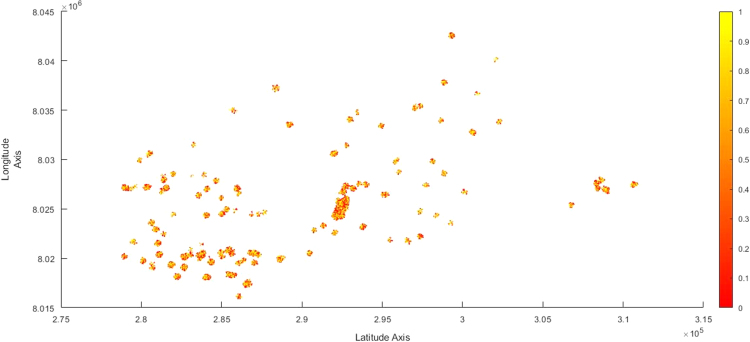
Scatterplot for Waypoints in Harare from 1990 to 2010.

The scatterplot clearly demonstrates that the IEEs heavily concentrated in all the five identified locational areas during the period (Fig. 5).

## 2 Experimental design, materials and methods

Spatial data was collected by means of geographic mapping of IEEs in the Central business district, suburban shopping centres, industrial centres, transportation and communication centres and homes as well as open spaces and along roads, using the global positioning system (GPS). The process started by capturing the oldest locations of IEEs, particularly the vegetables markets that were provided during the 1980s in low income residential areas. The period of location and the spatial spread was largely determined by the age of the infrastructure and the information that was supplied by city council officials that have worked for the two cities for over the 30 year period. A total of 1164 GPS points were captured in Harare. The base maps had earlier on been scanned and geo referenced before the actual identification and digitising process. Using a density analysis the changes of IEEs over time were calculated and the changes were expressed as absolute values and percentages.

### 2.1 The spatial spread of informal economic enterprises in the year 1990

**Fig. 6:**
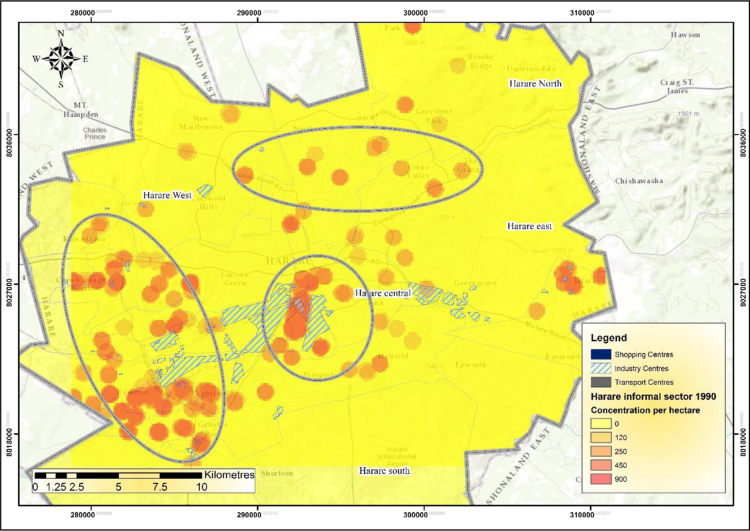
Density of informal economic enterprises in Harare during the year 1990.

Fig. 6 shows the density of IEEs in Harare during the 1990s. GIS point density analysis demonstrated that there were very few IEEs in the city in 1990 [7]. The density maps show striking locational patterns of IEEs with very dense concentrations of 900 IEEs/ha and low concentrations of 120/ha.

### 2.2 The spatial spread of informal economic enterprises in the year 2000

**Fig. 7:**
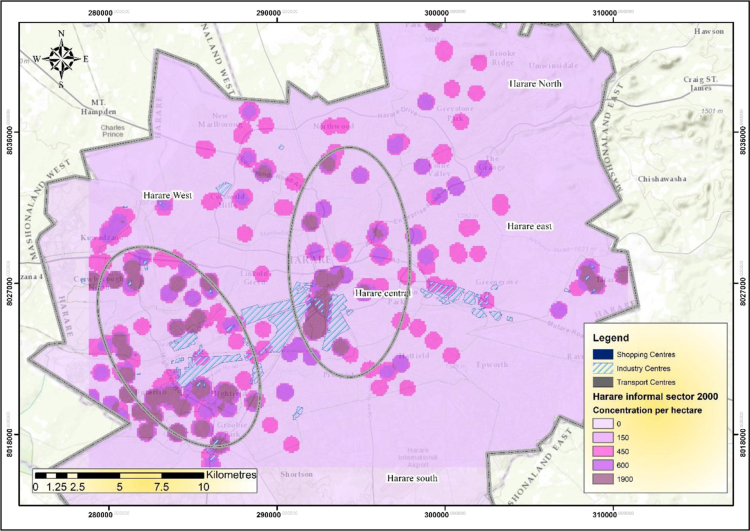
Density of informal economic enterprises in Harare in the year 2000.

GIS analysis revealed increased concentration of IEEs in residential areas and their spread in new places. According to Fig. 7, the concentration of informal businesses in Harare is very high in the case of trading and commerce activities that operate from very small spaces. The highest density of IEEs changed from 900 (IEEs/ha) in the 1980s to 1900 (IEEs/ha) during the 1990s, thus 111% change. The lowest concentration also changed from 120IEEs/ha to 150IEEs/ha, a low percentage change of 25% [8].

### 2.3 Spatial spread of informal economic enterprises in the year 2010

**Fig. 8:**
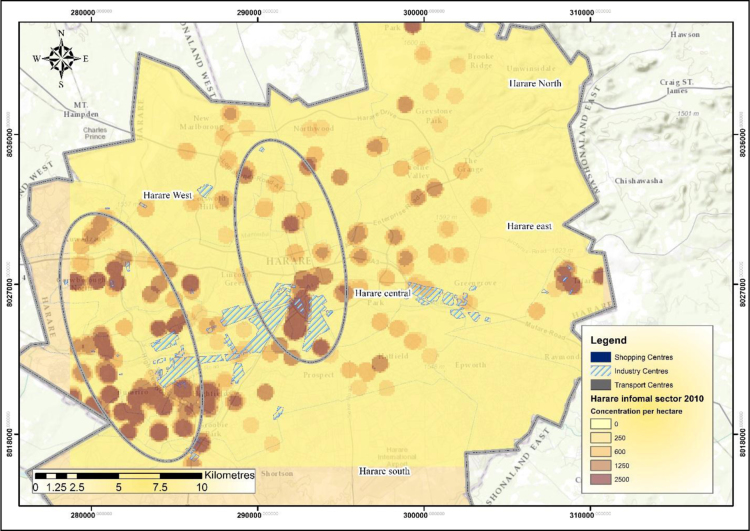
Density of informal economic enterprises in Harare in the year 2010.

GIS analysis reveals that the highest concentration of IEEs increased from 1900 IEEs/ha during the 1990s to 2500 IEEs during the 2000s whilst the lowest concentrations also changed from 150IEEs/ha to 250IEEs/ha (Fig. 8).

### 2.4 Combined spatial spread of formal economic enterprises during the period as a whole

**Fig. 9:**
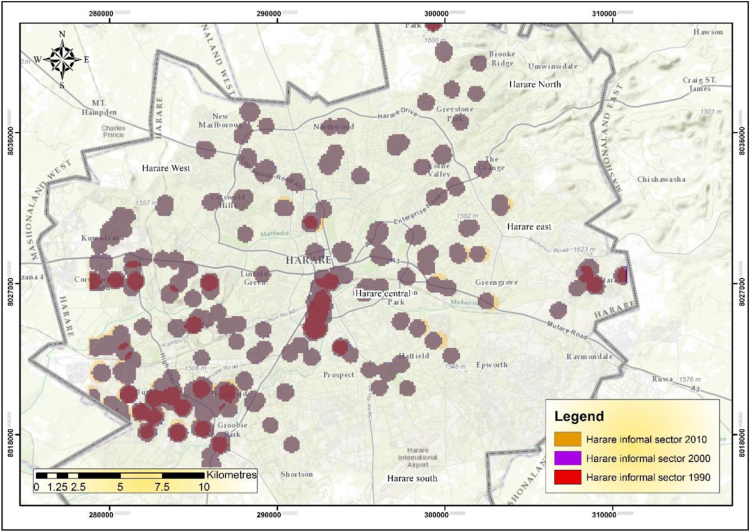
Cluster of informal economic enterprises in Harare during the period as a whole.

The IEEs increased during the period and their concentrations increased from 120IEEs/ha to 250IEEs/ha, that׳s denoting double growth (Fig. 9). It was found during the study that 4.7% of the FEEs are located in upmarket SSCs that include Semi Levy, Borrowdale Brooke, Westgate and High Glen in Harare. The invasion of CBDs prompted FEEs to leave, which in turn resulted in the CBDs losing firms to other functional areas of the city. It was found in the study that CBDs were host to 35.7% of the FEEs. The city centres accommodated 55.1% of the trade and commerce businesses, 27.2% of personal services, 8.4% of manufacturing and processing, 6.8% of transport and communication and 1.9% of construction enterprise in the FES [8].

As the traditional land zones such as the CBDs were invaded by the IEEs, 8.3% of the FEEs found refuge within suburbs adjacent to the city centres. Mostly medium density houses were converted to office use due to their proximity to the CBDs and environments conducive to doing businesses [8].
