# Supplementary material for: Mapping spatial locational trends of informal economic enterprises using mobile geographic information data in the city of in Harare, Zimbabwe
Source: Data Brief. 2018 Sep 18;20:1692–9. doi: 10.1016/j.dib.2018.09.037 (PMC6157609; doi:10.1016/j.dib.2018.09.037)
Supplement: Supplementary file 1 — Supplementary material [file mmc1.docx]

CONFLICT OF INTEREST

Dear Editor

This confirms that there is no conflict of interest around the authors and this study

Trynos and Inocent
